# First report of articuliths (free‐living geniculate corallines, Corallinales, Rhodophyta) in the northern hemisphere revealed during diversity surveys of Haida Gwaii, British Columbia, Canada

**DOI:** 10.1111/jpy.70049

**Published:** 2025-06-12

**Authors:** Keelie E. Taylor, Gary W. Saunders

**Affiliations:** ^1^ Centre for Environmental and Molecular Algal Research, Department of Biology University of New Brunswick Fredericton New Brunswick Canada; ^2^ Present address: Beaty Biodiversity Museum University of British Columbia Vancouver British Columbia Canada

**Keywords:** articuliths, British Columbia, DNA barcoding

## Abstract

Free‐living coralline beds are typically composed of rhodoliths, or unattached non‐geniculate coralline algae. In 2017, the first beds comprised primarily of free‐living geniculate coralline algae, termed articuliths, were documented in Arraial do Cabo Bay in southeastern Brazil. During routine barcode surveys of the Haida Gwaii, British Columbia, Canada flora, 16 rhodolith‐like specimens were collected from a rhodolith bed that DNA sequences assigned to the geniculate taxa *Calliarthron tuberculosum* and *Bossiella* sp. 1heteroforma. To our knowledge, articuliths have not been documented outside of Brazil; this discovery thus documents the first instance of northern hemisphere articuliths. Despite disparate gross morphologies to attached conspecific populations, anatomical observations revealed internal anatomies consistent with those of attached forms but with a significant reduction in the number of genicula and increased uniformity in intergenicular shape.

**Abbreviations**
BC: British Columbia
COI‐5P: cytochrome c oxidase subunit 1
PCR: polymerase chain reaction
*psb*A: photosystem II protein D1
*rbc*L: ribulose biphosphate carboxylase large subunit
UNB: University of New Brunswick

Although the global occurrence of rhodolith beds has been well established (Anderson et al., 2023; Foster, 2001), beds composed of articuliths, or unattached geniculate coralline algae, were first observed by Tâmega et al. (2017) in Southeastern Brazil. Four years later, these beds were revisited, and their composition reassessed to account for cryptic diversity (Tâmega et al., 2021). To our knowledge, articuliths have not been reported outside southeastern Brazil, with this study being the first report for the northern hemisphere.

During ongoing DNA barcode surveys of the British Columbia (BC) algal flora, specimens were collected via SCUBA from a rhodolith bed between Murchison and Faraday Islands in Haida Gwaii to assess the diversity of coralline species contributing to this bed (Table S1). Specimens were submitted as vouchers or photographic e‐vouchers to the Connell Memorial Herbarium at the University of New Brunswick (UNB; Thiers, 2025). Following Saunders and McDevit (2012), DNA was extracted from silica‐dried subsamples with subsequent polymerase chain reaction (PCR) amplification of the CO1‐5P, *rbc*L‐3P, and *psb*A gene markers (Saunders & Moore, 2013). For brightfield microscopy, specimens were decalcified in 5% acetic acid and sectioned using a Leica CM1850 freezing microtome. Vegetative structures were observed using a Leica CTR5000 microscope, and photomicrographs were taken using an OMAX A3514OU microscope‐mounted digital camera. For scanning electron microscopy, specimens were longitudinally fractured using a single‐edge razor blade and a hammer. Fractured material was mounted on aluminum stubs, coated in 28 nm of gold, and viewed with a JSM 6400 scanning microscope at an accelerating voltage of 15 kV.

Along with several non‐geniculate species (K. E. Taylor, G. W. Saunders, unpublished data), the *rbc*L‐3P and *psb*A gene sequence data assigned 16 unattached coralline collections to two geniculate species: *Calliarthron tuberculosum* (*n* = 14) and a novel species of *Bossiella* provisionally called *Bossiella* sp. 1heteroforma (*n* = 2), both of which had also been collected from the same region in an attached form (Table S1). *Calliarthron tuberculosum* articuliths were a 100% match in the *rbc*L‐3P and *psb*A genes to sequences for attached specimens, while *Bossiella* sp. 1heteroforma articuliths had 0% intraspecific variation but were 0.48% and 0.46% divergent from *Bossiella heteroforma* in *rbc*L‐3P and *psb*A sequences, respectively. COI‐5P was not successfully amplified for either the *C. tuberculosum* or *Bossiella* sp. 1heteroforma articuliths.

Attached *Calliarthron tuberculosum* had bead‐like basal intergenicula, the calcified segments of the thallus, that transitioned to a flattened/winged morphology in the medial portion of the erect axes (Figure 1a). Branch tips frequently terminated in smaller cylindrical segments. Genicula, the uncalcified joints between intergenicula, occurred at frequent/regular intervals along the axes and were externally visible. Additionally, genicula were always present where new branches diverged from the preceding intergenicular segment (Figure 1a). The unattached individuals of *C. tuberculosum* resembled their “branched” growth form typical of rhodoliths (Foster, 2001; Figure 1b). Genicula, while present, were few, occasionally corticated by calcified tissue and notably absent at branching points (Figure 1b,c). In articuliths, this reduction of genicula makes intergenicula appear elongate and frequently continuous at branching points. Intergenicular shape could best be described as cylindrical; however, due to their elongate nature, they were often more irregular or curved (Figure 1b,c) than what is typically seen in attached specimens of this species (Figure 1a).

**FIGURE 1:**
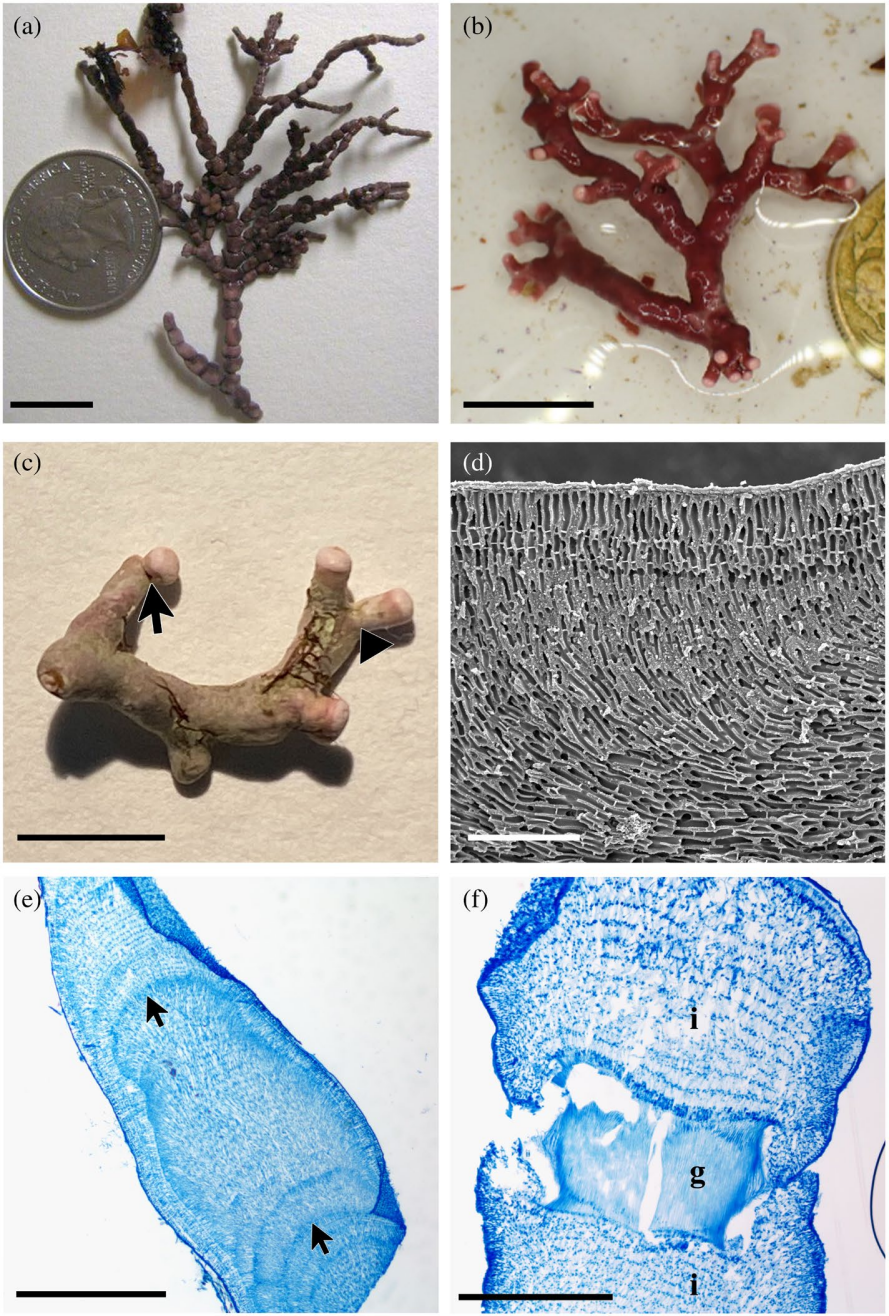
*Calliarthron tuberculosum* growth form variation and internal anatomy of articulith collections. (a) Attached morphology of *C. tuberculosum* with basal intergenicula broad to bead‐like and apical intergenicula cylindrical; genicula occur at regular intervals (UNB GWS021230, scale = 12.5 mm). (b) Articulith morphology of *C. tuberculosum* with elongated cylindrical intergenicula and fewer, irregularly spaced genicula (UNB GWS048575, scale = 12.5 mm). (c) Specimen showing branch tips with (arrow) and without (arrowhead) exposed genicula (UNB GWS048590, scale = 5 mm). (d) Longitudinal fracture of branch tip showing monomerous construction, elongate cell shape and cell fusions (UNB GWS048575, scale = 100 μm). (e) Longitudinal section of branch tip showing presence of growth bands (arrows) in medullary tissue (UNB GWS048590, scale = 1 mm). (f) Longitudinal section of branch tip showing a corticated geniculum (g) and adjacent intergenicula (i) (UNB GWS048575, scale = 500 μm).

Anatomically articulith specimens were consistent with those of attached *Calliarthron tuberculosum* despite their disparate external appearance (Figure 1b,c). Thallus construction was monomerous with elongate cells and interwoven medullary filaments, and cells of adjacent filaments were frequently joined by cell fusions (Figure 1d). Longitudinal sections of branches revealed growth bands similar to those typical of other rhodolith‐forming species (Figure 1e). Genicula, where present, were also of the typical morphology seen in *C. tuberculosum*, consisting of long, thin cells arranged in a single tier. However, in many cases, the genicula were corticated or covered by an outer layer of tissue and were only revealed upon sectioning (Figure 1f). The tissue covering the genicula was fragile and prone to tearing upon sectioning the decalcified specimen.

In addition to the two articulith specimens, three attached specimens of *Bossiella* sp. 1heteroforma were also collected from Haida Gwaii (Table S1). The attached specimens exhibited high variation in gross morphology, with the thalli densely branched and the branches consisting of flattened/winged intergenicula terminating in smaller cylindrical intergenicula (Figure 2a,b). Genicula occurred regularly along the axes and were externally visible. In unattached individuals, intergenicula were more commonly cylindrical throughout, with flattened segments restricted to where branches diverged (Figure 2c,d). Genicula were present but often occurred at wider‐spaced intervals, leading to occasional elongate intergenicula. Similar to unattached *Calliarthron tuberculosum*, some unattached *Bossiella* sp. 1heteroforma specimens also resembled the “branched” rhodolith morphology.

**FIGURE 2:**
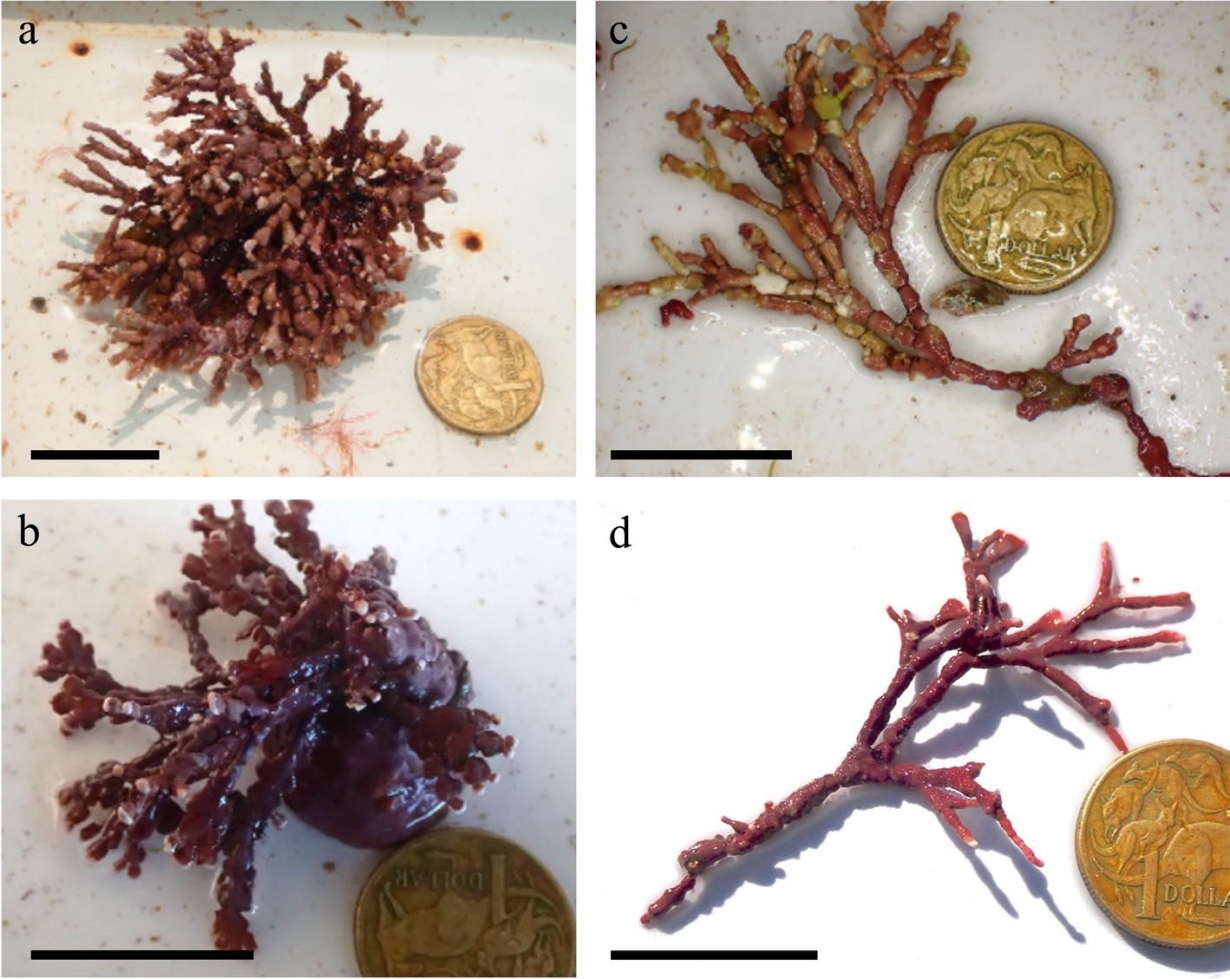
*Bossiella* sp. 1heteroforma growth form variation. (a–b) Attached specimens with flattened/winged intergenicula and branches terminating in short cylindrical segments (a, UNB GWS046399, scale = 25 mm; b, UNB GWS046829, scale = 25 mm). (c–d) Articulith specimens with typically cylindrical intergenicula, but flattened where multiple branches diverge, some intergenicula unusually elongate (c, UNB GW048593 scale = 25 mm; d, UNB GWS046756, scale = 25 mm).

In attached geniculate corallines, the genicula provide flexibility to upright fronds and reduce the drag forces resulting from the wave‐swept environments where they commonly grow (Denny & King, 2016; Janot & Martone, 2016, 2022). The articulith beds in Brazil have been reported to experience seasonal aggregation and dispersal related to winds, currents, and upwelling (Tâmega et al., 2021). However, the rhodolith beds in Haida Gwaii, where these new articuliths were observed, appeared more stable with conditions more typical of rhodolith beds, meaning they were sheltered from harsh waves outside of storms and seldom exposed to currents strong enough to dislodge individuals from the bed (Teichert et al., 2012; Millar & Gagnon, 2018; Lavenère‐Wanderley et al., 2021). This likely means that unattached individuals deposited into rhodolith beds are no longer subjected to high amounts of hydrodynamic strain. In members of *Calliarthron*, genicula form from specific medullary cells lengthening and decalcifying (Johansen, 1969). When fully formed, the remaining calcified cortical tissue around the joint breaks open to expose the geniculum (Martone, 2006). A reduction in the repetitive pulling and bending that would normally catalyze young genicula formation could result in these joints remaining encased by thin calcified tissue. It could also mean that as the fragment continues to increase in size, new genicula are not stimulated to form due to the lack of hydrodynamic pressures. As rhodolith branches tend to interlock, providing stability to beds, thallus rigidity owing to reduced genicula may even be favored as it decreases the likelihood of the unattached variety being dislodged by stronger currents or storms (Hinojosa‐Arango & Riosmena‐Rodríguez, 2004). This could also potentially correlate with the observed change in intergenicular shape and dimensions.

To our knowledge this is the first report of articuliths in the northern hemisphere. Although an exciting addition to the discovery of Tâmega et al. (2017), this phenomenon should be further researched with a focus on how articuliths interact with each other as well as the rhodoliths in these beds. Future research should also seek to identify additional populations of articuliths outside the channel between Murchison and Faraday Island, as well as the possibility that species in addition to *Calliarthron tuberculosum*, *Bossiella* sp. 1heteroforma, and those described by Tâmega et al. (2021) are contributing to these rhodolith/articulith beds.

## AUTHOR CONTRIBUTIONS

**Keelie E. Taylor:** Data curation (equal); formal analysis (equal); funding acquisition (supporting); investigation (equal); methodology (equal); writing – original draft (lead); writing – review and editing (equal). **Gary W. Saunders:** Conceptualization (lead); data curation (equal); formal analysis (equal); funding acquisition (lead); investigation (equal); methodology (equal); resources (lead); writing – review and editing (equal).

## Supporting information


**Table S1.** Collection data and GenBank accession numbers for articulith and attached collections of *Bossiella heteroforma*, *Bossiella* sp. 1heteroforma, and *Calliarthron tuberculosum*. Specimens deposited in the Connell Memorial Herbarium (UNB; Thiers, 2025).
