# Supplementary material for: First report of articuliths (free‐living geniculate corallines, Corallinales, Rhodophyta) in the northern hemisphere revealed during diversity surveys of Haida Gwaii, British Columbia, Canada
Source: J Phycol. 2025 Jun 12;61(4):1038–42. doi: 10.1111/jpy.70049 (PMC12351355; doi:10.1111/jpy.70049)
Supplement: Supplementary file 1 — Table S1. Collection data and GenBank accession numbers for articulith and attached collections of Bossiella heteroforma, Bossiella sp. 1heteroforma, and Calliarthron tuberculosum. Specimens deposited in the Connell Memorial Herbarium (UNB; Thiers, 2025). [file JPY-61-1038-s001.docx]

**Table S1**

| **Taxon name** | **Herbarium ID** | **Collection data** | **GenBank acc. No. (COI-5P)** | **GenBank acc. No. (*rbc*L-3P)** | **GenBank acc. No. (*rbc*L)** | **Genbank acc. No. (*psb*A)** |
| --- | --- | --- | --- | --- | --- | --- |
| *Bossiella heteroforma* | GWS008716 | Subtidal (18 m) on rock, Bamfield, Seapool Rock, British Columbia, Canada, 48.816 -125.209, 16.6.2007, G.W. Saunders, B. Clarkston, D. McDevit & K. Roy | HM918814 |  | KJ592005 | KJ637829 |
| *Bossiella heteroforma* | GWS010087 | Subtidal (17 m) on rock, Tahsis, Island south of Clotchman I., Spanish Pilot Group, British Columbia, Canada, 49.6145 -126.583, 23.5.2008, K. Hind & D. McDevit | KJ591790 |  | KJ592006 | KJ637828 |
| *Bossiella heteroforma* | GWS010088 | Subtidal (17 m) on rock, Tahsis, Island south of Clotchman I., Spanish Pilot Group, British Columbia, Canada, 49.6145 -126.583, 23.5.2008, K. Hind & D. McDevit | KJ591789 |  |  |  |
| *Bossiella heteroforma* | GWS022338 | Subtidal (16 m) on shale, Monterey Bay (shale bed), California, United States of America, 36.609 -121.879, 23.5.2010, B. Clarkston, K. Hind & S. Toews | HQ544243 |  |  |  |
| *Bossiella heteroforma* | GWS028231 | Subtidal (6 m) on rock, Haswell Bay (north point in bay), Gwaii Haanas, Haida Gwaii, British Columbia, Canada, 52.529 -131.607, 8.7.2011, G.W. Saunders & K. Dixon | KJ637745 | PQ883164 |  | PQ883178 |
| *Bossiella heteroforma* | GWS044674 | Subtidal (4 m) on rock, between Wiah Point & Cape Edenshaw (#3), NW of Masset, Haida Gwaii, British Columbia, Canada, 54.1121 -132.345, 5.8.2018, G.W. Saunders & C. Brooks |  | PQ883163 |  |  |
| *Bossiella heteroforma* | GWS045452 | Subtidal (2.5 m) on rock, Faraday I., N Site, Plot 1 (5-10 ft), Gwaii Hanaas, Haida Gwaii, British Columbia, Canada, 52.6152 -131.465, 18.7.2019, G.W. Saunders & C. Brooks |  |  |  |  |
| *Bossiella* sp. 1heteroforma | GWS046282 | Subtidal (10 m) on rock, articulated, Murchison I. NW site, Plot 1 (10-30 ft), Gwaii Haanas, British Columbia, Canada, 52.61456 -131.44443, 16.7.2019, G.W. Saunders & C. Brooks |  | PQ883156 |  |  |
| *Bossiella* sp. 1heteroforma | GWS046399 | Subtidal (8 m) on worm tube, articulated, Faraday I., N Site, Plot 1 (10-30 ft), Gwaii Haanas, British Columbia, Canada, 52.61523 -131.46466, 18.7.2019, G.W. Saunders & C. Brooks |  | PQ883150 |  |  |
| *Bossiella* sp. 1heteroforma | GWS046756 | Subtidal (7 m), articulith, channel btw Murchison & Faraday I., Gwaii Haanas, British Columbia, Canada, 52.59703 -131.47345, 27.7.2022, G.W. Saunders & C. Brooks |  | PQ883167 |  | PQ883180 |
| *Bossiella* sp. 1heteroforma | GWS046829 | Subtidal (10 m) on limpet, articulated, Murchison I. NW site, Plot 2 (10-30 ft) Gwaii Haanas, British Columbia, Canada, 52.60913 -131.46582, 28.7.2022, G.W. Saunders & C. Brooks |  | PQ883160 |  |  |
| *Bossiella* sp. 1heteroforma | GWS048593 | Subtidal (5 m), articulith, channel btw Murchison & Faraday I., Gwaii Haanas, British Columbia, Canada, 52.59687 -131.47513, 2.8.2022, G.W. Saunders & C. Brooks |  | PQ883153 |  |  |
| *Calliarthron tuberculosum* | GWS002904 | Low intertidal on rock pools in channel past Blowhole, articulated, Bamfield, Blowhole at Brady’s Beach, British Columbia, Canada, 48.8235 -125.1613, 8.6.2005, G.W. Saunders | PQ883022 |  | PQ883151 | PQ883173 |
| *Calliarthron tuberculosum* | GWS046750 | Subtidal (7 m), articulith, channel btw Murchison & Faraday I., Gwaii Haanas, British Columbia, Canada, 52.59703 -131.47345, 27.7.2022, G.W. Saunders & C. Brooks |  |  |  | PQ883177 |
| *Calliarthron tuberculosum* | GWS046751 | Subtidal (7 m), articulith, channel btw Murchison & Faraday I., Gwaii Haanas, British Columbia, Canada, 52.59703 -131.47345, 27.7.2022, G.W. Saunders & C. Brooks |  | PQ883162 |  | PQ883176 |
| *Calliarthron tuberculosum* | GWS046752 | Subtidal (7 m), articulith, channel btw Murchison & Faraday I., Gwaii Haanas, British Columbia, Canada, 52.59703 -131.47345, 27.7.2022, G.W. Saunders & C. Brooks |  | PQ883165 |  | PQ883179 |
| *Calliarthron tuberculosum* | GWS046753 | Subtidal (7 m), articulith, channel btw Murchison & Faraday I., Gwaii Haanas, British Columbia, Canada, 52.59703 -131.47345, 27.7.2022, G.W. Saunders & C. Brooks |  | PQ883168 |  | PQ883181 |
| *Calliarthron tuberculosum* | GWS046754 | Subtidal (7 m), articulith, channel btw Murchison & Faraday I., Gwaii Haanas, British Columbia, Canada, 52.59703 -131.47345, 27.7.2022, G.W. Saunders & C. Brooks |  | PQ883154 |  | PQ883174 |
| *Calliarthron tuberculosum* | GWS046755 | Subtidal (7 m), articulith, channel btw Murchison & Faraday I., Gwaii Haanas, British Columbia, Canada, 52.59703 -131.47345, 27.7.2022, G.W. Saunders & C. Brooks |  | PQ883159 |  | PQ883175 |
| *Calliarthron tuberculosum* | GWS048575 | Subtidal (5 m), articulith, channel btw Murchison & Faraday I., Gwaii Haanas, British Columbia, Canada, 52.59687 -131.47513, 2.8.2022, G.W. Saunders & C. Brooks |  | PQ883161 |  |  |
| *Calliarthron tuberculosum* | GWS048589 | Subtidal (5 m), articulith, channel btw Murchison & Faraday I., Gwaii Haanas, British Columbia, Canada, 52.59687 -131.47513, 2.8.2022, G.W. Saunders & C. Brooks |  | PQ883169 |  |  |
| *Calliarthron tuberculosum* | GWS048590 | Subtidal (5 m), articulith, channel btw Murchison & Faraday I., Gwaii Haanas, British Columbia, Canada, 52.59687 -131.47513, 2.8.2022, G.W. Saunders & C. Brooks |  | PQ883158 |  |  |
| *Calliarthron tuberculosum* | GWS048594 | Subtidal (5 m), articulith, channel btw Murchison & Faraday I., Gwaii Haanas, British Columbia, Canada, 52.59687 -131.47513, 2.8.2022, G.W. Saunders & C. Brooks |  | PQ883170 |  |  |
| *Calliarthron tuberculosum* | GWS048602 | Subtidal (5 m), articulith, channel btw Murchison & Faraday I., Gwaii Haanas, British Columbia, Canada, 52.59687 -131.47513, 2.8.2022, G.W. Saunders & C. Brooks |  | PQ883152 |  |  |
| *Calliarthron tuberculosum* | GWS048603 | Subtidal (5 m), articulith, channel btw Murchison & Faraday I., Gwaii Haanas, British Columbia, Canada, 52.59687 -131.47513, 2.8.2022, G.W. Saunders & C. Brooks |  | PQ883157 |  |  |
| *Calliarthron tuberculosum* | GWS048608 | Subtidal (5 m), articulith, channel btw Murchison & Faraday I., Gwaii Haanas, British Columbia, Canada, 52.59687 -131.47513, 2.8.2022, G.W. Saunders & C. Brooks |  | PQ883155 |  |  |
| *Calliarthron tuberculosum* | GWS048616 | Subtidal (5 m), articulith, channel btw Murchison & Faraday I., Gwaii Haanas, British Columbia, Canada, 52.59687 -131.47513, 2.8.2022, G.W. Saunders & C. Brooks |  | PQ883166 |  |  |
